# Loss of hepatic DEPTOR alters the metabolic transition to fasting

**DOI:** 10.1016/j.molmet.2017.02.005

**Published:** 2017-02-17

**Authors:** Alexandre Caron, Mathilde Mouchiroud, Nicolas Gautier, Sébastien M. Labbé, Romain Villot, Laurie Turcotte, Blandine Secco, Guillaume Lamoureux, Michael Shum, Yves Gélinas, André Marette, Denis Richard, David M. Sabatini, Mathieu Laplante

**Affiliations:** 1 Centre de recherche de l'Institut universitaire de cardiologie et de pneumologie de Québec (CRIUCPQ), Faculté de Médecine, Université Laval, 2725 Chemin Ste-Foy, Québec, Qc, G1V 4G5, Canada; 2 Whitehead Institute for Biomedical Research, Nine Cambridge Center, Cambridge, MA, 02142, USA; 3 Howard Hughes Medical Institute, Department of Biology, Massachusetts Institute of Technology, Cambridge, MA, 02139, USA; 4 Koch Center for Integrative Cancer Research at MIT, 77 Massachusetts Avenue, Cambridge, MA, 02139, USA

**Keywords:** DEPTOR, mTOR, Liver, Glucose, Fasting

## Abstract

**Objective:**

The mechanistic target of rapamycin (mTOR) is a serine/threonine kinase that functions into distinct protein complexes (mTORC1 and mTORC2) that regulates growth and metabolism. DEP-domain containing mTOR-interacting protein (DEPTOR) is part of these complexes and is known to reduce their activity. Whether DEPTOR loss affects metabolism and organismal growth *in vivo* has never been tested.

**Methods:**

We have generated a conditional transgenic mouse allowing the tissue-specific deletion of DEPTOR. This model was crossed with CMV-cre mice or Albumin-cre mice to generate either whole-body or liver-specific DEPTOR knockout (KO) mice.

**Results:**

Whole-body DEPTOR KO mice are viable, fertile, normal in size, and do not display any gross physical and metabolic abnormalities. To circumvent possible compensatory mechanisms linked to the early and systemic loss of DEPTOR, we have deleted DEPTOR specifically in the liver, a tissue in which DEPTOR protein is expressed and affected in response to mTOR activation. Liver-specific DEPTOR null mice showed a reduction in circulating glucose upon fasting versus control mice. This effect was not associated with change in hepatic gluconeogenesis potential but was linked to a sustained reduction in circulating glucose during insulin tolerance tests. In addition to the reduction in glycemia, liver-specific DEPTOR KO mice had reduced hepatic glycogen content when fasted. We showed that loss of DEPTOR cell-autonomously increased oxidative metabolism in hepatocytes, an effect associated with increased cytochrome c expression but independent of changes in mitochondrial content or in the expression of genes controlling oxidative metabolism. We found that liver-specific DEPTOR KO mice showed sustained mTORC1 activation upon fasting, and that acute treatment with rapamycin was sufficient to normalize glycemia in these mice.

**Conclusion:**

We propose a model in which hepatic DEPTOR accelerates the inhibition of mTORC1 during the transition to fasting to adjust metabolism to the nutritional status.

## Highlights

• Whole-body DEPTOR KO mice are viable and do not display abnormalities.
• Liver-specific DEPTOR KO mice are hypoglycemic when fasted.
• Loss of DEPTOR promotes mTORC1 and increases oxidative metabolism.
• Rapamycin corrects hypoglycemia in liver-specific DEPTOR KO mice.

## 1 Introduction

The mechanistic target of rapamycin complex 1 (mTORC1) is an evolutionary conserved serine/threonine kinase that plays important roles in regulating cell metabolism, growth, and proliferation [1]. In response to nutrients and growth factors, mTORC1 activates several processes including protein and lipid synthesis. Studies indicate that mTORC1 is a key sensor allowing cells and tissues to adapt their metabolism in response to nutritional cues [1]. mTORC1 regulates glucose and lipid metabolism by controlling various metabolic processes in several organs, including the liver. In this tissue, mTORC1 controls ketogenesis [2], lipogenesis [3], [4], [5], [6], glucose metabolism [6], and mitochondrial dynamics [7] to ensure an appropriate feeding-to-fasting transition.

Because mTORC1 controls several energy-consuming processes, its activity must be tightly regulated to couple anabolism to the nutritional state. In response to growth factors (insulin), a cascade of intracellular events allows phosphatidylinositol 3-kinase (PI3K) to promote the activity of downstream kinases including protein kinase B (Akt/PKB). When active, Akt/PKB phosphorylates and inhibits the tuberous sclerosis complex (TSC), which acts as a GTPase-activating protein (GAP) for the small GTPase Ras-homolog enriched in brain (Rheb) [8], [9], [10]. In its GTP-bound state, Rheb stimulates mTORC1 kinase activity [11]. In parallel, amino acids activate mTORC1 independently of PI3K by promoting the translocation of mTORC1 to the lysosomal surface, where Rheb resides [12]. The recruitment of mTORC1 on the lysosome, a prerequisite for mTORC1 activation, involves several proteins including the Rag GTPase (RagA, B, C and D) [12], the Ragulator complex [13], v-ATPase [14], and folliculin [15].

In addition to these upstream regulators, the core proteins composing mTORC1 also includes modulators of mTORC1 activity, namely DEP domain-containing mTOR-interacting protein (DEPTOR) and proline-rich Akt substrate of 40 kDa (PRAS40) [16], [17], [18]. The role of these proteins in regulating mTORC1 action has been well demonstrated *in vitro*. Overexpression of both DEPTOR and PRAS40 in cells repress mTORC1 activity, mimicking a state of low nutrients and growth factors [16], [17], [18]. Despite these findings, the roles of these proteins *in vivo* are still poorly understood, especially because diverging observations were made using different approaches and models. For example, peripheral overexpression of DEPTOR promotes diet-induced obesity [19] whereas its overexpression in the mediobasal hypothalamus protects mice against body weight gain [20]. Likewise, liver-specific overexpression of PRAS40 or systemic ablation of this protein both improve glucose homeostasis in mice [21], [22]. These results indicate that the functions of these proteins likely differ between cell types and tissues. In this context, there is a clear need to develop conditional mouse models to specifically dissect the role of these proteins in specific cell populations.

To address these important issues, we developed a novel conditional knockout mouse for DEPTOR. Here, we report that whole-body deletion of DEPTOR does not lead to any apparent metabolic disturbances. Both male and female mice lacking DEPTOR are viable, fertile, normal in size, and metabolically healthy. Because prenatal DEPTOR deletion may have led to early developmental compensation inherently masking the resulting phenotype, we have deleted DEPTOR specifically in the liver, a tissue in which DEPTOR protein is expressed and regulated in response to mTORC1 activation. Using two different approaches, we show that postnatal liver-specific ablation of DEPTOR reduces circulating glucose upon fasting. This effect was not associated with changes in hepatic glucose production but was linked to a sustained reduction in circulating glucose levels during insulin tolerance tests. Importantly, mice lacking DEPTOR specifically in liver had reduced hepatic glycogen content when fasted, together with enhanced oxidative metabolism in hepatocytes. Sustained mTORC1 activation upon fasting was responsible for the resulting altered metabolic transition to fasting, as acute treatment with rapamycin, a specific mTORC1 inhibitor, was sufficient to normalize glycemia. Together, these results indicate that DEPTOR plays an important role in liver feeding-to-fasting transition by ensuring an optimal inhibition of mTORC1 when nutrients are low.

## 2 Material and methods

### 2.1 Animal care

Animal care and handling were performed in accordance with the Canadian Guide for the Care and Use of Laboratory Animals and all experimental procedures received prior approval from the Laval University Animal Care Committee (CPAUL). Male mice were maintained on a 12-hour light/dark cycle (lights on 0600–1800), while individually housed in ventilated cages at an ambient temperature of 23 ± 1 °C.

### 2.2 Mouse model generation

For generation of *Deptor*^LoxP/LoxP^ mice, a BAC clone (identifier: RP24-167K18, strain: C57BL/6J) containing *Deptor* locus was obtained from the RPCI-24 mouse genomic DNA library [23]. Standard PCR and cloning procedures were used to generate 5′ homology arm of 4.2 kb and a 3′ homology arm of 2.4 kb. These arms were cloned into PGKneoF2L2DTA [24] vector and inserted to surround a fragment of 852 bp containing the exon 2 of *Deptor*. In the final targeting construct, the neomycin resistance (neo) cassette was flanked LoxP sites and Flp recognition target (Frt) recombination sites. *Deptor* exon 2 was flanked by 5′ and 3′ LoxP sites. The targeting vector was linearized and electroporated into embryonic stem cells derived from 129/SvEv mice [25]. Clones were analyzed for correct integration by Southern blot. The following primers were used to generate a 519 bp probe to screen for positive integration (5′-CCT AGC ACA TAG GGC GAC TCT CA-3′; 5′-CAA GGC CTC AAT TCG ATG CTT-3′). This procedure was performed on genomic DNA that was treated overnight with BamHI (New England Biolabs, R0136). Positive clones were also validated by PCR. Chimeric mice were obtained by microinjection of the correctly targeted clones into BALB/C blastocysts and crossed with C57BL/6J mice to obtain offspring with germ line transmission. The mice analyzed in this study were backcrossed to C57BL/6J. Some of the backcrosses involved mice constitutively expressing the FLP recombinase so as to excise the neomycin cassette from the targeted allele [26]. All the studies were performed on littermates with the same genetic background. For the systemic or liver-specific invalidation of DEPTOR using Cre mice, mice were bred to homozygosity for the *Deptor*^LoxP^ allele and then crossed to mice expressing the Cre-recombinase transgene from the constitutively expressed CMV promoter (The Jackson Laboratory, Stock 006054) [27] or from the liver-specific albumin promoter (The Jackson Laboratory, Stock 003574) [28]. In the studies described here, Alb-Li-*Deptor*^wt^ and Alb-Li-*Deptor*^ko^ mice were of the following genotype Alb-cre^−/−^; Deptor^LoxP/LoxP^ and Alb-cre^+/?^; Deptor^LoxP/LoxP^ respectively. In all these studies, mice were backcrossed to C57BL/6J for at least 6 generations. For liver-specific recombination of the *Deptor*^LoxP^ allele in adult mice, 100 μl of high-titre Ad5 (empty or cre) (3–6) × 10^10^ PFU ml^−1^ was administered via retro-orbital injection under isoflurane anaesthesia. These studies were performed on *Deptor*^LoxP/LoxP^ mice backcrossed for 3 generations.

### 2.3 Genotyping

PCR genotyping of *Deptor*^LoxP/LoxP^ mice was performed with the following primers: *Deptor* forward = 5′-TGG CAC TGT CAA GGT GTG GG-3′, *Deptor* Δ485 = 5′-AAG TCA CCA GCC TGG AAG TG-3′, *Deptor* reverse = 5′-GAT GCG ATT GGG AAG AGA TGA G-3′. Using these primers, an amplicon of 310 bp is produced corresponding to the wild-type *Deptor* allele. A fragment of 358 bp is produced corresponding to the *Deptor*^LoxP^ allele. An amplicon of 485 bp is detected when the exon 2 is removed upon Cre recombination.

### 2.4 Glucose, insulin and pyruvate tolerance tests

For the GTT, mice were fasted 16 h (from 1600 to 0800) and were injected ip with 1 g/kg of d-Glucose (Sigma Aldrich, St Louis, MO). For the ITT, animals were fasted for 6 h (from 0800 to 1400) and were injected ip with 0.75 U/kg of insulin (Humulin, Lilly, Canada). For the PTT, mice were fasted for 16 h (from 1600 to 0800) and were injected ip with 1 mg/kg sodium pyruvate (Sigma Aldrich). Blood samples were collected from the tail vein and glucose was measured using a glucometer (Roche, Accu-Chek Performa).

### 2.5 Plasma metabolite measurement

Blood was collected using syringes conditioned with EDTA. Plasma was stored at −80 °C for further biochemical analyses. Plasma metabolites were measured using the following commercial kits; insulin (EMD Millipore, SRI-13K), triglycerides (Thermo Fisher Scientific, TR22421), NEFAs (Wako, 999-34691, 995-34791, 991-34891, 993-35191, 276-76491), and FGF-21 (R&D Systems, MF2100). Circulating glucose levels were measured from tail blood using an instant glucometer (Roche, Accu-Chek Performa).

### 2.6 PET procedure

All *in vivo* PET experiments were initiated immediately after the insertion of a cannula in the tail vein for an injection of ^11^C-acetate PET tracer. All imaging experiments were performed on the avalanche photodiode-based small animal PET scanner (LabPET/Triumph, Gamma Medica, Northridge, CA) of the Sherbrooke Molecular Imaging Centre, having a 7.5 cm axial field-of-view. The animals were anaesthetized with isoflurane (1.75%, 1.5 L/min) delivered through a nose cone and placed in the supine position on the scanner bed. A bolus of ^11^C-acetate (10 MBq, in 0.2 ml of 0.9% NaCl) was injected via the caudal vein over 30 s after starting PET data acquisition. A 20-min dynamic data acquisition with ^11^C-acetate was done to determine liver blood flow and oxidative metabolism. Low dose μCT scans were obtained using a Gamma Medica Triumph X-O small-animal CT scanner composed of a 40 W X-ray tube with a 75 μm focal spot diameter and a 2240 × 2368 CsI flat panel X-ray detector. The detector pixel size was 50 μm, and a 2 × 2 pixels binning scheme was used. Scans were performed at 60 kVp and 230 μA using 512 projections in fly mode to reduce exposure. For ^11^C-acetate PET images, a dynamic series of 28 frames (1 × 30, 12 × 10, 8 × 30, 6 × 90, and 1 × 300 s) were sorted out, and 3D images were reconstructed using 15 iterations of a maximum-likelihood expectation-maximization (MLEM) algorithm incorporating physical description of the detector response function. Regions of interest (ROIs) were drawn on short-axis images and confirmed with the μCT scan. Tissue time-activity curves were generated by those ROI drawn on liver. Input curves (blood time-activity curve) were extracted by means of a ROI drawn on the left ventricular cavity blood pool in summed last-frame images to seek better contrast. The sizes of these almost-circular ROIs were compared with images of eight cylinders of different diameters from which a recovery factor was extracted and applied to the ROIs for partial volume correction [29].

### 2.7 Measurement of glucose uptake by tissues

^3^H-deoxyglucose ([1,2-^3^H(N)]-Deoxy-d-glucose) was used to evaluate glucose uptake in inguinal white adipose tissue (iWAT), epididymal WAT (eWAT), brown adipose tissue (BAT), heart, gastrocnemius muscle, and spleen, as previously described [30]. The tracer (Moravek Biochemicals, Inc. Brea, CA, USA) was dissolved in normal saline supplemented with 4% bovine serum albumin (BSA). In a first set of experiment, mice received an intraperitoneal bolus of 10 μCi of ^3^H-deoxyglucose in a total volume of 150 μl following a 11 h fast (from 2000 to 0700). Four hours following the injection, mice were euthanized with an overdose of anesthetic and tissues were collected, weighed, and homogenized. In a second set of experiment, mice were fasted for 6 h (from 0800 to 1400), injected with insulin (0.75 U/kg) and a bolus of 10 μCi of ^3^H-deoxyglucose, and euthanized with an overdose of anesthetic 4 h later. Specific fractional uptake of ^3^H-deoxyglucose was determined using a scintillation counter (liquid scintillation analyzer Tri-Carb 2900TR, PerkinElmer, Montreal, QC, Canada), as previously described [31]. Uptake data were expressed as the percentage of injected dose of [3H] per gram of tissues.

### 2.8 Hepatic glycogen content extraction and measurement

Glycogen was measured in liver samples as described [32]. Briefly, glycogen was extracted in 30% KOH saturated with Na_2_SO_4_, precipitated in 95% ethanol, and re-suspended in distilled H_2_O. After addition of phenol and H_2_SO_4_, absorbance at 490 nm was measured in triplicates.

### 2.9 Hepatic triglyceride content extraction and measurement

Liver sections (25–50 mg) were homogenized in 900 μl of a 2:1 chloroform:methanol mixture. The homogenate was combined with 300 μl of methanol, vortexed, and centrifuged for 15 min at 3,000 rpm. 825 μl of supernatant was transferred to a new glass tube, 400 μl of chloroform and 275 μl of 0.73% NaCl was added, and the resulting mixture was vortexed for 30 s and centrifuged at 5,000 rpm for 3 min. The upper phase was discarded and 800 μl of a 3/48/47 mixture of chloroform:methanol:NaCl (0.58%) was added to wash the lower phase and was centrifuged at 5,000 rpm for 3 min. After three washes, the lower phase was evaporated and resuspended in 1 ml of fresh isopropanol. Triglyceride levels were determined with a standard assay kit (Thermo Fisher Scientific, TR22421) according to the manufacturer's instructions.

### 2.10 Cell culture

AML12 cells were purchased from ATCC (CRL-2254) and cultured in a 1:1 mixture of Dulbecco's modified Eagle's medium and Ham's F12 medium with 0.005 mg/ml insulin, 0.005 mg/ml transferrin, 5 ng/ml selenium, and 40 ng/ml dexamethasone, 90%; fetal bovine serum, 10%, as prescribed.

### 2.11 Virus preparation and cell infection

The vectors used to knock down RFP or DEPTOR are: sh_RFP: TRCN0000072203; shDeptor_1: TRCN0000110157; shDeptor_2: TRCN0000110159. The sequence of these hairpins can be retrieved on the RNAi Consortium (TRC) website http://www.broadinstitute.org/rnai/public/gene/search. These vectors were co-transfected with the Delta VPR envelope and CMV VSV-G [33]. Virus-containing supernatants were collected at 48 h after transfection and filtered using a 0.45μ filter. Cells were infected for 24 h in the presence of 8 μg/ml polybrene. After infection, the cells were dispersed into fresh medium. Cells were selected on the following days with 1 μg/ml puromycin.

### 2.12 Seahorse experiments

Functional mitochondrial measurements were performed using the Seahorse XF Cell Mito Stress Test according to manufacturer's instructions. Briefly, AML12 cells were seeded onto an XF24 Cell Culture Microplate (Agilent Seahorse Bioscience) at 3,000 cells/well and incubated at 37 °C overnight in regular media. The following morning, regular media was replaced for Seahorse XF Base Medium containing 1 mM pyruvate (Gibco, 11360070), 2 mM l-glutamine (Gibco, 25030149) and 25 mM glucose (Sigma, G8270). Cell metabolic rates were measured using an XF24 Extracellular Flux Analyzer (Agilent Seahorse Bioscience) following the sequential addition of 1 μM oligomycin (oligo; Sigma 75351; port A), 4 μM carbonyl cyanide 4-(trifluoromethoxy)-phenylhydrazone (FCCP; Sigma, C2920; Port B) and 0.5 μM antimycin A (AA; Sigma, A8674; Port C) + 0.5 μM rotenone (rot; Sigma, R8875; Port C). Oxygen consumption rate (OCR) was normalized to protein concentration using the Protein Assay reagent (Bio-Rad, 500-0006) and expressed as % from baseline. Non-glycolysis acidification (NGA), glycolysis (G), glycolysis capacity (GC) and glycolytic reserve (GR), were also evaluated using the Seahorse XF Glycolysis Stress Test according to manufacturer's instructions. Briefly, AML12 cells were seeded onto an XF24 Cell Culture Microplate (Agilent Seahorse Bioscience) at 3,000 cells/well and incubated at 37 °C overnight in regular media. The following morning, regular media was replaced for Seahorse XF Base Medium containing 1 mM l-glutamine (Sigma), and cells were incubated for one hour at 37 °C in a non-CO_2_ incubator. Extracellular acidification rate (ECAR) was measured following the sequential addition of 10 mM glucose (Sigma, G8270; port A), 1 μM oligomycin (oligo; Sigma, 75351; port B), and 50 mM 2-DG (Sigma, D6134; port C).

### 2.13 Measurement of mitochondrial DNA content

Mitochondrial DNA (mtDNA) content was measured similarly as described before [34], [35], [36]. Briefly, total DNA was isolated using a commercial kit DNeasy blood and tissue kit (Qiagen, 69504). Ten ng of total DNA was used to amplify mtDNA-encoded NADH dehydrogenase I (ND1) and nuclear DNA-encoded Beta-actin using real-time PCR. The following primers were used to amplify ND1 (5′-ACA AAC ACT TAT TAC AAC CCA AGA A-3′; 5′-TTA GGG CGT TTA TTA GAA TAA TGT T-3′) and Beta-Actin (5′-AGC GGA CAC TGG CAC AGC CAA CTT-3′; 5′-AAA TGC CCA CAC CGC GAC CCT A-3′). Relative mtDNA content was calculated from the ratio of ND1 to genomic Beta-actin.

### 2.14 Quantitative real-time PCR

Total mRNA was isolated from tissues using the RNeasy Lipid Tissue Mini Kit (Qiagen, 74104). Total mRNA was isolated from cells using E.Z.N.A.^®^ Total RNA Kit I (Omega Bio-Tek, R6834-02). The RNA concentrations were estimated from absorbance at 260 nm. cDNA synthesis was performed using the iScript™ Advanced cDNA Synthesis Kit for RT-qPCR (Bio-Rad). mRNA extraction and cDNA synthesis were performed following the manufacturer's instructions. cDNA was diluted in DNase-free water (1:15) before quantification by real-time PCR. mRNA transcript levels were measured in duplicate samples using a CFX96 or CFX384 touch™ real-time PCR (Bio-Rad, Mississauga, ON, Canada). Chemical detection of the PCR products was achieved with SYBR Green (Bio-Rad, 172-5271). At the end of each run, melt curve analyses were performed, and representative samples of each experimental group were run on agarose gel to ensure the specificity of the amplification. Gene expression was corrected for the expression level of reference genes (*Arbp*). The following primers were used:

**Table:** 

Gene	Forward primer	Reverse primer
*Arbp*	5′-AGAAACTGCTGCCTCACATC-3′	5′-CATCACTCAGAATTTCAATGG-3′
*Cs*	5′-AAGTTGGCAAAGACGTGTCA-3′	5′-CCGAGACACTCCAAACAGGAC-3′
*Cytc*	5′-TGGACCAAATCTCCACGGTCTGTT-3′	5′-TAGGTCTGCCCTTTCTCCCTTCTT-3′
*Fgf21*	5′-CAGCCCTGATGGAATGGATGAGAT-3′	5′-GAGGCTTTGACACCCAGGATTTGA-3′
*G6pc*	5′-GAAGGCCAAGAGATGGTGTGA-3′	5′-TGCAGCTCTTGCGGTACATG-3′
*G6pdx*	5′-GATGTCTTCTGTGGGAGCCA-3′	5′-CCTATGGTCCCAAAGTCCTTCC-3′
*Gaa*	5′-GTCACTGGACAGGGGATGTG-3′	5′-AGGTAGGGCAGAAGGGCATA-3′
*Gapdh*	5′-GGCAAATTCAACGGCACAGT-3′	5′-CTCGTGGTTCACACCCATCA-3′
*Glut1*	5′-CACTGTGGTGTCGCTGTTTG-3′	5′-AAAGATGGCCACGATGCTCA-3′
*Glut2*	5′-ACCTTGGCTTTCACTGTCTTCACT-3′	5′-ATTCCGCCTACTGCAAAGCT-3′
*Gpi1*	5′-TGGAGAAACTCTTGCCACACA-3′	5′-GAATCATGGGAGGTCACAGCA-3′
*Gys*	5′-CGCCGAATTGGCCTTTTCAA-3′	5′-AAGCTGAGGGATCTGCGATG-3′
*Hk1*	5′-ACCTGAATGTAACCGTGGGC-3′	5′-GCTCTTAGGCGTTCGTAGGG-3′
*Ldha*	5′-GCAGTGGAAGGAGGTTCACAA-3′	5′-GAAGTGCTAGGACACGGGGA-3′
*Pcx*	5′-CTGGCTGTAAGCGACCTGAA-3′	5′-CTTAGCCACCTTGTCCCCTG-3′
*Pdha*	5′-ACACAGCATGAGTGACCCTG-3′	5′-CACCCATCCACCCACCTAAC-3′
*Pdk1*	5′-TCCTTAGAGGGCTACGGGAC-3′	5′-GCTTCCAGGCGGCTTTATTG-3′
*Pdk2*	5′-GTACTCCACAGCTCCCACAC-3′	5′-ATTCAGAGGTGGCAGCATCC-3′
*Pdk4*	5′-CAAGGCATCCTGGAGTATAAAG-3′	5′-CAAAGGCATCTTGGACTACTG-3′
*Pepck*	5′-GAGTGGAGACCGCAGGAC-3′	5′-CAGGTATTTGCCGAAGTTGTAG-3′
*Pfkfb3*	5′-ATCCCACGGGAGAGTCCTAC-3′	5′-GACAGTGTGGAGCGGACATT-3′
*Pfkm*	5′-GATGGGTGTGGAAGCAGTGA-3′	5′-ATTCATCACGGCCACTGTGT-3′
*Pklr*	5′-GGCAGATGATGTGGACCGAA-3′	5′-AAAGGGGAGAGGCGTTTCAG-3′
*Pkm*	5′-TCTGTACCGTGGCATCTTCC-3′	5′-GCCCAGAGTGAGCACTACAA-3′
*Ppara*	5′-AGAGCCCCATCTGTCCTCTC-3′	5′-ACTGGTAGTCTGAAAACCAAA-3′
*Tkt*	5′-ATGAGGATTTCCAGGTCGGC-3′	5′-CACTGCCTCTCCTATGCCAC-3′

### 2.15 Western blotting

All cells were rinsed twice with ice-cold PBS before lysis. Cells were lysed with Triton-X 100 containing lysis buffer (50 mM HEPES, pH 7.4, 2 mM EDTA, 10 mM sodium pyrophosphate, 10 mM sodium glycerophosphate, 40 mM NaCl, 50 mM NaF, 2 mM sodium orthovanadate, 1% Triton-X 100, and one tablet of EDTA-free protease inhibitors Roche per 25 ml). Tissues were homogenized with the same buffer supplemented with 0.1% sodium lauryl sulfate and 1% sodium deoxycholate. Cells and tissues were rotated at 4 °C for 10 min and then the soluble fractions of cell lysates were isolated by centrifugation at maximum speed for 10 min in a microcentrifuge. Protein levels were then quantified using Bradford reagents and analyzed by Western blotting. Samples were loaded on 4–12% Nupage precast gels or 10% Tris-Glycine gels (Life Technologies). Proteins were transferred to PVDF membranes blocked in 5% milk diluted in PBS-Tween and incubated with their primary antibody overnight at 4 °C. The following antibodies were used: Akt (Cell Signaling Technology, 4691, dilution 1:1000), phospho-AKT S473 (Cell Signaling Technology, 9271, dilution 1:1000), S6 (Cell Signaling Technology, 2217, dilution 1:2500), phospho-S6 S240/244 (Cell Signaling Technology, 5364, dilution 1:1000), DEPTOR (Novus Biologicals, NBP1-49674, lot no. A-2, dilution 1:1000), and mitochondrial complexes I to V (Abcam, 110413, dilution 1:250). Secondary antibodies were purchased from Cell Signaling Technology (7076S and 7074S) and diluted 1:5000. Amersham ECL Western Blotting Detection Reagent (RPN2106) was used to image the blots.

### 2.16 Statistical analyses

Data are expressed as mean ± SEM. Comparisons between two experimental conditions were analyzed by Student's unpaired *t* test. Two-way ANOVA was used to compare more than two experimental conditions. All statistical tests were performed using GraphPad Prism (version 6.0c), and p < 0.05 was considered statistically significant.

## 3 Results

### 3.1 Whole-body DEPTOR knockout mice are viable, fertile, normal in size, and do not display any gross physical or metabolic abnormalities

**Figure 1:**
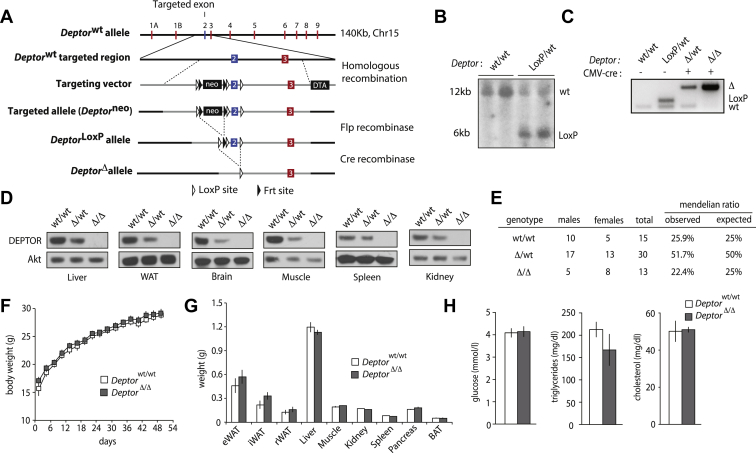
**Whole-body DEPTOR null mice are viable, fertile, normal in size, and do not display any gross physical abnormalities.** (A) Overview of the strategy developed to delete *Deptor* in mice. The exon 2 of *Deptor* was floxed with LoxP sites using a targeting vector containing Diphtheria Toxin A and Neomycin resistance cassettes. Positive ES cells carrying the targeted allele (*Deptor*^Neo^) were injected into blastocysts to produce chimeric mice. Following germ line transmission, mice carrying the *Deptor*^Neo^ allele were crossed with mice expressing the Flp recombinase to eliminate the Neo cassette, thus creating the *Deptor*^LoxP^ allele. When crossed with mice expressing the Cre recombinase, the LoxP sites surrounding the exon 2 of *Deptor* recombine, which disrupts *Deptor* coding sequence, by introducing premature STOP codons. (B) Southern blot performed on targeted ES cells confirming the insertion of the targeting vector in the *Deptor* locus. (C) Genotyping validation showing the recombination of *Deptor*^LoxP^ allele in wild-type mice or CMV-cre mice. (D) Western blot showing the expression of DEPTOR in tissues of *Deptor* wild-type (wt/wt), heterozygote (Δ/wt) or knockout (Δ/Δ) mice. Akt was used as a loading control. (E) Mendelian ratios calculated from the crossing of *Deptor* heterozygote (Δ/wt) mice. (F) Body weight and (G) tissue weight of whole-body *Deptor* wild-type and knockout mice. The data are expressed as the mean ± SEM for n = 7–8. (H) Blood metabolites measured in *Deptor* wild-type or knockout mice. Blood was collected from mice that were fasted overnight. The data are expressed as the mean ± SEM for n = 7–8.

In order to investigate the importance of DEPTOR in regulating physiological processes *in vivo*, we have floxed *Deptor* allele by homologous recombination. An overview of the strategy and the validation of our approach are depicted in Figure 1A,B. Briefly, the second exon of *Deptor*, which is common to the two *Deptor* isoforms, was flanked with LoxP sites to produce *Deptor*^LoxP/LoxP^ mice. Loss of this exon disrupts *Deptor* reading frame and leads to the insertion of premature stop codons. To determine whether DEPTOR is required for normal mouse development and growth, *Deptor*^LoxP/LoxP^ mice were first crossed with mice expressing the Cre recombinase under the control of a human cytomegalovirus (CMV) minimal promoter (CMV-Cre) (Figure 1C). In this strain, the Cre gene is expressed early during embryogenesis and recombination of LoxP sites occurs in all tissues [27]. Because the recombination is germinal, CMV-cre *Deptor*^LoxP/LoxP^ mice can transmit a knockout (KO) allele to their progeny. Here, this allele is defined as the *Deptor* Δ allele. As shown in Figure 1D, this strategy was efficient to eliminate DEPTOR in all the tissues tested. DEPTOR KO mice are viable and born at the expected Mendelian ratio (Figure 1E). These mice are normal and do not show change in body and organ weights (Figure 1F–G and Supplementary Figure 1A–B). Moreover, we did not detect significant changes in plasma glucose and lipid levels in DEPTOR KO mice (Figure 1H and Supplementary Figure 1C). Together, these results indicate that the chronic and systemic deletion of DEPTOR has minimal impacts in mice. Whether the absence of phenotype in whole-body DEPTOR null mice is due to compensatory mechanisms secondary to the chronic loss of the protein is possible.

### 3.2 Liver-specific DEPTOR knockout mice exhibit lower blood glucose upon fasting

**Figure 2:**
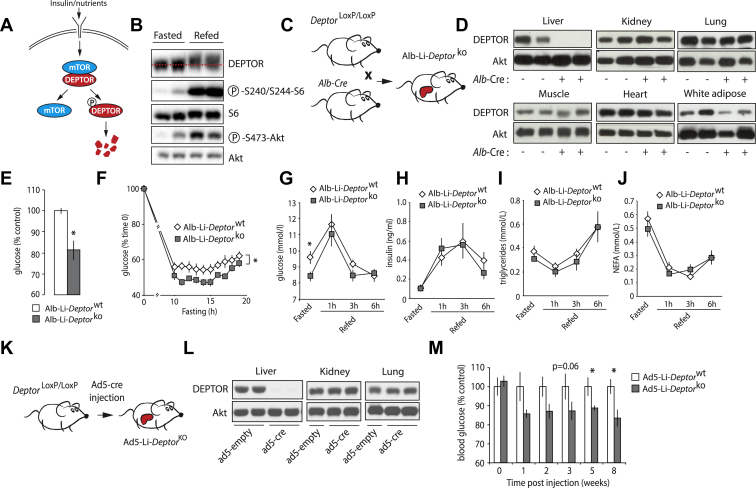
**Liver-specific DEPTOR null mice exhibit lower blood glucose levels when fasted.** (A) Schematic representation of the regulatory process controlling DEPTOR protein levels in cells. (B) Western blot analysis showing the impact of fasting and refeeding on DEPTOR in the liver. All mice were fasted for 14 h. One group was sacrificed in fasting condition (Fasted) and the other was given free access to food for 6 h following fasting (Refed). (C) Schematic representation of the strategy used to produce Alb-Li-*Deptor*^wt^ and Alb-Li-*Deptor*^ko^ mice. (D) Western blot showing the expression of DEPTOR in tissues of Alb-Li-*Deptor*^wt^ and Alb-Li-*Deptor*^ko^ mice. Akt was used as a loading control. (E) Glycemia measured in fasted Alb-Li-*Deptor*^wt^ and Alb-Li-*Deptor*^ko^ mice. Mice were fasted for 12 h. Results are presented as percentage of control. The data are expressed as the mean ± SEM for n = 5. * indicates p < 0.05 versus Alb-Li-*Deptor*^wt^ mice. (F) Blood glucose levels measured in fasted Alb-Li-*Deptor*^wt^ and Alb-Li-*Deptor*^ko^ mice. Mice were fasted and glucose levels were measured over time. Results are presented as percentage initial blood glucose measure before fasting. The data are expressed as the mean ± SEM for n = 9. * indicates p < 0.05 versus Alb-Li-*Deptor*^wt^ mice. (G to J) Blood glucose, insulin, triglycerides, and NEFA measured Alb-Li-*Deptor*^wt^ and Alb-Li-*Deptor*^ko^ mice. Mice were fasted for 12 h and then refed a normal chow diet for 1, 3, or 6 h. The data are expressed as the mean ± SEM for n = 5. * indicates p < 0.05 versus Alb-Li-*Deptor*^wt^ mice. (K) Schematic representation of the strategy used to produce Ad5-Li-*Deptor*^wt^ and Ad5-Li-*Deptor*^ko^ mice. (L) Western blot showing the expression of DEPTOR in tissues of Ad5-Li-*Deptor*^wt^ and Ad5-Li-*Deptor*^ko^ mice. Akt was used as a loading control. (M) Glycemia measured in Ad5-Li-*Deptor*^wt^ and Ad5-Li-*Deptor*^ko^ mice fasted for 10 h. Results are presented as percentage of control. The data are expressed as the mean ± SEM for n = 8–9. * indicates p < 0.05 versus Ad5-Li-*Deptor*^wt^ mice.

When active, mTOR phosphorylates DEPTOR, which promotes their dissociation and the degradation of DEPTOR by the proteasome (Figure 2A) [16], [37], [38], [39]. Because DEPTOR is heavily phosphorylated upon mTOR activation, a clear mobility shift in DEPTOR migration can be observed by SDS-PAGE [16]. Although, this phenomenon has been confirmed several times *in vitro*, it is unknown whether DEPTOR is similarly regulated *in vivo*. In response to feeding, insulin and nutrients rapidly turn on mTORC1 and mTORC2 in the liver [2]. As shown in Figure 2B, activation of mTOR signaling by feeding induced a clear mobility shift in DEPTOR protein levels, confirming that the protein is subject to a similar regulation in cancer cells and in normal tissues.

Because mTOR signaling plays key metabolic roles in the liver, and because DEPTOR protein is affected in this tissue in response to nutritional status, we have deleted DEPTOR in hepatocytes by crossing *Deptor*^LoxP/LoxP^ mice with mice expressing the Cre recombinase under the control of *Albumin* promoter (Alb-cre) (Figure 2C). Since Alb-Cre is expressed later in development than CMV-Cre [27], [40], we reasoned that this strategy could limit possible compensatory mechanisms linked to the chronic loss of DEPTOR. As shown in Figure 2D, this approach was efficient to specifically delete DEPTOR in the liver. From now, we will refer to these mice as the Alb-Li-*Deptor*^ko^ mice. As reported above with the whole-body DEPTOR KO mouse, Alb-Li-*Deptor*^ko^ mice did not show change in body weight (Supplementary Figure 2A). Specifically, liver size was the same between Alb-Li-*Deptor*^wt^ and Alb-Li-*Deptor*^ko^ mice (Supplementary Figure 2B). Interestingly, we observed that Alb-Li-*Deptor*^ko^ mice had reduced fasting blood glucose levels compared to Alb-Li-*Deptor*^wt^ mice (Figure 2E–F). This effect was specific to glucose and not associated with changes in circulating insulin, triglycerides or non-esterified fatty acids (NEFA) between the genotypes (Supplementary Figure 2C–E). Importantly, the reduction in fasting glycemia was transient in Alb-Li-*Deptor*^ko^ mice. Hence, over a long fasting period, the difference between Alb-Li-*Deptor*^wt^ and Alb-Li-*Deptor*^ko^ tended to disappear, suggesting that DEPTOR loss alters the transition, but not the systemic adaptation to fasting (Supplemental Figure 2F). Supporting a specific role for DEPTOR in regulating metabolism during fasting, we observed that glucose levels were normalized when Alb-Li-*Deptor*^ko^ mice were refed (Figure 2G). Circulating insulin, triglycerides and NEFA were also normal in these mice upon refeeding (Figure 2H–J). Food intake was not different between Alb-Li-*Deptor*^wt^ and Alb-Li-*Deptor*^ko^ mice (Supplementary Figure 2G).

To confirm the acute role of DEPTOR in regulating fasting glucose levels in mice, we have injected adult *Deptor*^LoxP/LoxP^ mice with type 5 adenoviruses encoding Cre recombinase (Ad5-cre) (Figure 2K). As shown before, this strategy is efficient to specifically eliminate floxed alleles in the liver of adult mice [2]. A validation of this approach is shown in Figure 2L. From now, we will refer to these mice as the Ad5-Li-*Deptor*^ko^ mice. As expected, losing hepatic DEPTOR did not affect body and liver weight (Supplementary Figure 2H–I). Confirming the importance of DEPTOR in regulating fasting glycemia, we observed a progressive reduction in circulating glucose in Ad5-Li-*Deptor*^ko^ mice (Figure 2M). Altogether, these results suggest a key role for DEPTOR in regulating fasting glucose levels.

### 3.3 Liver-specific DEPTOR loss alters systemic glucose homeostasis

**Figure 3:**
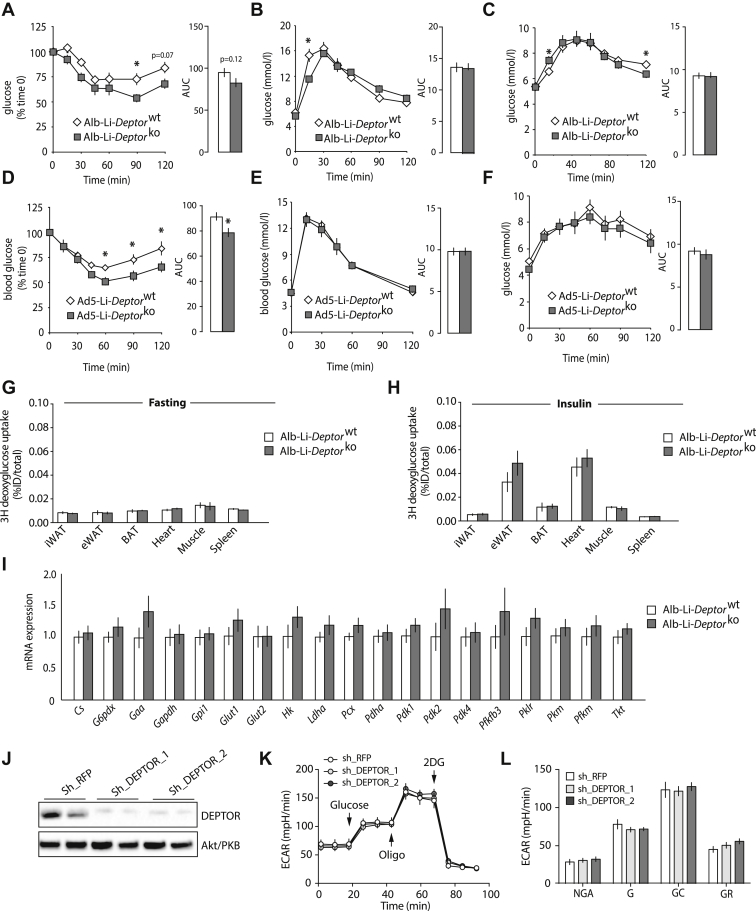
**Liver-specific DEPTOR loss alters systemic glucose homeostasis.** (A) Insulin, (B) glucose, and (C) pyruvate tolerance tests performed in Alb-Li-*Deptor*^wt^ and Alb-Li-*Deptor*^ko^ mice. The data are expressed as the mean ± SEM for n = 6–9. * indicates p < 0.05 versus Alb-Li-*Deptor*^wt^ mice. (D) Insulin, (E) glucose, and (F) pyruvate tolerance tests performed in Ad5-Li-*Deptor*^wt^ and Ad5-Li-*Deptor*^ko^ mice. The data are expressed as the mean ± SEM for n = 7–9. * indicates p < 0.05 versus Ad5-Li-*Deptor*^wt^ mice. For all the graphs presented from A to F, the area under the curve (AUC) is presented. ^3^H-deoxyglucose uptake by tissues in (G) fasted (11 h) or (H) insulin-injected Alb-Li-*Deptor*^wt^ and Alb-Li-*Deptor*^ko^ mice. The data are expressed as the mean ± SEM for n = 5. (I) Quantitative RT-PCR analyses of liver samples isolated from fasting Alb-Li-*Deptor*^wt^ and Alb-Li-*Deptor*^ko^ mice. The data are expressed as the mean ± SEM for n = 7–9. (J) Western blot showing the expression of DEPTOR in AML12 cells. Akt was used as a loading control. (K) Extracellular acidification rate (ECAR) in AML12 cells following the sequential addition of glucose (10 mM), oligomycin (oligo; 1 μM), and 2-DG (100 mM). The data are expressed as the mean ± SEM for n = 3–4 per condition. (L) Non-glycolysis acidification (NGA), glycolysis (G), glycolysis capacity (GC), and glycolytic reserve (GR) calculated from the ECAR presented in K.

To better define the impact of hepatic DEPTOR deletion on systemic glucose homeostasis, we have performed insulin, glucose, and pyruvate tolerance tests (ITT, GTT and PTT, respectively) in Alb-Li-*Deptor*^ko^ and Ad5-Li-*Deptor*^ko^ mice and their wild-type counterparts. First, mice were subjected to an ITT to measure the impact of hepatic DEPTOR loss on insulin sensitivity. As shown in Figure 3A,D, both Alb-Li-*Deptor*^ko^ and Ad5-Li-*Deptor*^ko^ showed a reduction in circulating glucose compared to control mice following the intraperitoneal injection of insulin. Mice were next subjected to GTT to assess glucose tolerance. As shown in Figure 3B,E, we did not observe a consistent effect of DEPTOR on circulating glucose levels during this test. Finally, to determine whether the reduction in fasting glucose levels was linked to a lower capacity for gluconeogenesis, mice were subjected to a PTT. This test did not reveal any striking differences between Alb-Li-*Deptor*^ko^ or Ad5-Li-*Deptor*^ko^ and their respective controls (Figure 3C,F). Supporting these results, we did not observe changes in the expression of key gluconeogenic genes in the liver of DEPTOR null mice (Supplementary Figure 3A). It is interesting that we did not observe a significant reduction in fasting blood glucose levels between control and liver-specific DEPTOR null mice prior to initiate GTT and PTT (See time 0, Figure 3B–C and E–F). This is likely explained by the fact that mice were subjected to a long fasting prior to these tests. As shown in Supplemental Figure 2F, the difference in glycemia between control and liver-specific DEPTOR null mice disappears upon an extended fasting period.

To determine whether the reduction in glycemia found in response to DEPTOR loss was associated with change in glucose uptake by tissues, Alb-Li-*Deptor*^wt^ and Alb-Li-*Deptor*^ko^ mice were injected with ^3^H-deoxyglucose ([1,2-^3^H(N)]-deoxy-d-glucose), a non-metabolizable glucose analogue that remains trapped in most tissues following its uptake. Mice were injected with ^3^H-deoxyglucose in the fasting state or following an acute insulin injection. As shown in Figure 3G–H, the loss of hepatic DEPTOR did not significantly affect glucose uptake by tissues in both fasted and insulin-injected mice. The liver is one of the few tissues that can mobilize ^3^H-deoxyglucose following its uptake [30], thus preventing the use of ^3^H-deoxyglucose to evaluate hepatic glucose uptake. To circumvent this limitation, we measured the expression of glucose transporters and several enzymes controlling glycolysis in the liver of Alb-Li-*Deptor*^wt^ and Alb-Li-*Deptor*^ko^ mice. As shown in Figure 3I, we did not observe significant difference in the expression of all these genes. Supporting these results, knocking down DEPTOR in mouse AML12 hepatocytes did not affect any of the key parameters of glycolytic flux, including glycolytic capacity (Figure 3J–L). The results presented above indicate that peripheral glucose uptake and gluconeogenesis are unlikely to play a major role in the reduction of blood glucose levels in Alb-Li-*Deptor*^ko^ mice.

### 3.4 Loss of DEPTOR promotes oxidative metabolism in hepatocytes

**Figure 4:**
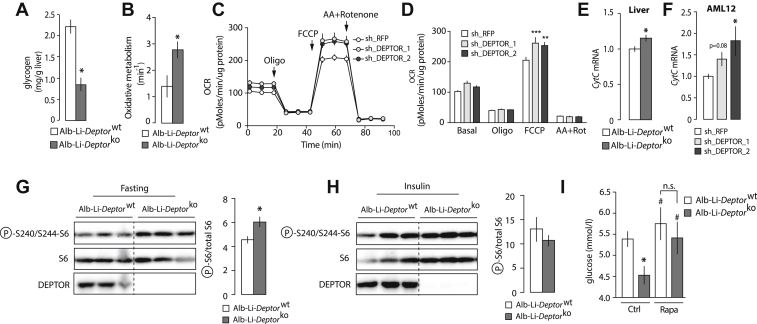
**DEPTOR loss promotes oxidative metabolism and mTORC1 activity.** (A) Hepatic glycogen content in Alb-Li-*Deptor*^wt^ and Alb-Li-*Deptor*^ko^ mice. Mice were fasted for 12 h and sacrificed. The data are expressed as the mean ± SEM for n = 5. * indicates p < 0.05 versus Alb-Li-*Deptor*^wt^ mice. (B) Oxidative activity index measured by PET in the liver of Alb-Li-*Deptor*^wt^ and Alb-Li-*Deptor*^ko^ mice. Mice were fasted before the injection of ^11^C-acetate. The data are expressed as the mean ± SEM for n = 4. * indicates p < 0.05 versus Alb-Li-*Deptor*^wt^ mice. (C–D) Oxygen consumption rate (OCR) in AML12 cells following the sequential addition of oligomycin (oligo; 1 μM), FCCP (4 μM) and antimycin A + rotenone (0.5 μM). The data were normalized to protein concentration and are expressed as the mean ± SEM for n = 3–4 per condition. (E–F) *CytC* mRNA expression measured by quantitative RT-PCR analyses in (E) liver samples isolated from fasting Alb-Li-*Deptor*^wt^ and Alb-Li-*Deptor*^ko^ mice or (F) AML12 cells in which DEPTOR was knocked down. The data are expressed as the mean ± SEM for n = 7–9 for the liver sample and n = 4–5 for AML12 cells. * indicates p < 0.05 versus control. (G–H) Western blot showing the impact of DEPTOR loss on the phosphorylation of S6 in (G) fasted mice (12 h) or (H) mice injected with insulin (3.8 IU/Kg, 5 min). Representative samples are shown. Quantifications of the blots are shown on the right side of each panel. The data are expressed as the mean ± SEM for n = 3–9. * indicates p < 0.05 versus control. (I) Impact of rapamycin on circulating glucose in Alb-Li-*Deptor*^wt^ and Alb-Li-*Deptor*^ko^ mice. Mice were injected with rapamycin (2 mg/kg) and then fasted overnight. Glucose levels were measured the next day. The data are expressed as the mean ± SEM for n = 6–7. * indicates p < 0.05 versus Alb-Li-*Deptor*^wt^ and # indicates p < 0.05 versus Alb-Li-*Deptor*^ko^ injected with PBS.

It is well established that hepatic glycogen is an important source of glucose that is mobilized over the first hours of fasting to maintain blood glucose levels [41]. Because the impact of DEPTOR on fasting glycemia was transient and occurred only during the first phase of food deprivation, we hypothesized that DEPTOR deletion might have reduced glycemia by delaying glycogen mobilization by the liver. According to this model, a rise in glycogen content was expected in the liver of Alb-Li-*Deptor*^ko^ mice. Surprisingly, not only did Alb-Li-*Deptor*^ko^ mice not exhibit elevated hepatic glycogen content compared to control mice, but they showed lower hepatic glycogen deposition (Figure 4A). Supporting a cell-autonomous role for DEPTOR in regulating glycogen metabolism in hepatocytes, Alb-Li-*Deptor*^ko^ mice did not show alteration in glycogen deposition in skeletal muscle (Supplementary Figure 4A). We also observed that hepatic glycogen levels were rapidly normalized when Alb-Li-*Deptor*^ko^ mice were given access to food (Supplementary Figure 4B), indicating that DEPTOR loss unlikely reduced hepatic glycogen accumulation by impairing its synthesis. Supporting this result, the expression of *Glycogen synthase* (*Gys*) was not affected by DEPTOR loss (Supplementary Figure 4C). In addition to entering in glycogen synthesis, glucose molecules also serve as key substrate to support lipogenesis [42]. To test whether DEPTOR loss altered systemic glucose homeostasis and glycogen deposition by preferentially promoting lipogenesis, we measured hepatic triglyceride levels in Alb-Li-*Deptor*^wt^ and Alb-Li-*Deptor*^ko^ mice. As shown in Supplemental Figure 4D, DEPTOR deletion did not affect liver triglyceride levels.

As described above, Alb-Li-*Deptor*^ko^ mice were hypoglycemic when fasted, a phenomenon that occurred concomitantly with a reduction in hepatic glycogen deposition. Together, these results indicate that hepatic and circulating glucose levels are reduced upon hepatic DEPTOR deletion. To determine whether hepatic DEPTOR could affect circulating glucose levels and hepatic glycogen deposition by promoting glucose oxidation in the liver, we performed positron emission tomography experiments using ^11^C-acetate, a metabolic tracer that allows live measurement of oxidative metabolism by the tricarboxylic acid (TCA) cycle [9], [14], [41]. Time-activity curves calculated from ^11^C-acetate dynamic scans revealed an elevation in metabolic activity in the liver of fasted Alb-Li-*Deptor*^ko^ mice (Figure 4B). Interestingly, knocking down DEPTOR in mouse AML12 hepatocytes increased mitochondrial maximal respiration (Figure 4C–D). These results indicate that DEPTOR depletion drives liver oxidative metabolism in a hepatocyte-autonomous fashion. Importantly, this rise in oxidative metabolism measured in the liver of Alb-Li-*Deptor*^ko^ mice and AML12 cells depleted from DEPTOR was not a consequence of increased in mitochondrial content or in expression of proteins of the respiratory chain (Supplementary Figure 4E–H). Nonetheless, we measured elevations in *Cytc* expression in response to DEPTOR depletion (Figure 4E–F), an effect that may have contributed to promote oxidative metabolism.

### 3.5 Rapamycin corrects fasting blood glucose in Alb-Li-*Deptor*^ko^ mice

To define whether DEPTOR loss affects circulating glucose levels during the transition to fasting by acutely modulating mTOR activity, liver samples were collected from fasted and insulin-injected Alb-Li-*Deptor*^wt^ and Alb-Li-*Deptor*^ko^ mice. Consistent with a role for DEPTOR in inhibiting mTORC1, we observed a rise in the phosphorylation of the ribosomal protein S6 in the liver of fasted Alb-Li-*Deptor*^ko^ mice (Figure 4G). DEPTOR loss did not affect the phosphorylation of AKT on Ser473 and Thr308, which are residues targeted by mTORC2 and phosphoinositide-dependent kinase-1 (PDK1) respectively (data not shown). Interestingly, we found that the elevation in S6 phosphorylation in the liver of Alb-Li-*Deptor*^ko^ mice was detected only in fasted mice. As shown in Figure 4H, no difference on S6 phosphorylation was observed in the liver of Alb-Li-*Deptor*^wt^ and Alb-Li-*Deptor*^ko^ mice injected with insulin. These results indicate that DEPTOR represses mTORC1 only in conditions of food deprivation. To determine whether sustained mTORC1 activation during fasting participates in the deregulation of glycemia in Alb-Li-*Deptor*^ko^ mice, mice were injected with a single dose of the mTORC1 inhibitor rapamycin prior to food deprivation. Although rapamycin did not affect glycemia in control animals, it normalized fasting blood glucose levels in Alb-Li-*Deptor*^ko^ mice (Figure 4I). These results indicate that the reduction in circulating glucose observed in response to hepatic DEPTOR loss is mediated by mTORC1. We propose a model in which hepatic DEPTOR accelerates the inhibition of mTORC1 during the transition to fasting to adjust metabolism to the nutritional status.

## 4 Discussion

DEPTOR was recently discovered as an mTOR-interacting protein whose phosphorylation is very sensitive to mTOR activation [16]. The phosphorylation of DEPTOR promotes its dissociation from mTOR and favors its degradation by the proteasome [16], [37], [38], [39]. Because DEPTOR depletion activates and its overexpression represses mTOR activity, DEPTOR was characterized as an endogenous inhibitor of both mTORC1 and mTORC2 [16]. However, recent works performed in mice have revealed that the impact of DEPTOR on mTOR signaling is complex and may differ between cell types and tissues [19], [20], [43], [44].

Here, we have developed genetic mouse models to dissect the role of DEPTOR at the systemic and hepatocyte-specific levels. We first show that whole-body deletion of DEPTOR does not affect viability, fertility, growth and metabolism in male and female mice. Although the absence of phenotype may sound surprising based on the pleiotropic actions of mTOR signaling [1], prenatal DEPTOR deletion may have led to early developmental compensation, as often observed in several knockout mouse models [45]. For example, it is possible that DEPTOR loss may have been compensated by the upregulation of other negative regulators of the pathway, such as PRAS40. Considering this important issue, we have deleted DEPTOR later in development and specifically in the liver, a key metabolic tissue in which DEPTOR protein is expressed and regulated in response to mTORC1 activation. We found that postnatal hepatocyte-specific ablation of DEPTOR reduces circulating glucose during fasting, an effect that is rapidly lost upon re-feeding. Importantly, hepatic loss of DEPTOR did not affect the expression of gluconeogenic genes and the production of glucose during the PTT. However, glycogen content was reduced and oxidative metabolism increased in fasted mice lacking DEPTOR. We found that sustained mTORC1 activation upon fasting was responsible for the altered metabolic transition to fasting, as acute treatment with the mTORC1 inhibitor rapamycin was sufficient to normalize glycemia. Together, these results indicate that DEPTOR plays an important role in liver feeding-to-fasting transition by ensuring the optimal inhibition of mTORC1 when nutrients are low.

In the liver, mTORC1 regulates several metabolic processes including ketogenesis [2], lipogenesis [3], [4], [5], [6], glucose metabolism [6] and mitochondrial dynamics [7]. Importantly, liver transition to fasting is characterized by a reduction in oxidative metabolism [6], [7]. In fact, the disengagement of mTORC1 during the transition to fasting reduces the overall respiratory capacity of liver mitochondria [7]. The fact that mice lacking DEPTOR exhibit increased hepatic oxidative metabolism during fasting supports the idea that DEPTOR is required to fully disengage mTORC1 when energy is low. In the absence of DEPTOR, mTORC1 remains active, which promotes oxidative metabolism, increases glycogen depletion and reduces circulating glucose. Supporting this model, we found that DEPTOR deletion promotes S6 phosphorylation during fasting and that the mTORC1 inhibitor rapamycin was sufficient to fully rescue fasting hypoglycemia in mice lacking DEPTOR. These results are in line with previous observations showing that neonate knock-in mice that express a constitutively active form of RagA, an essential mediator of amino acid signals to mTORC1, exhibit a pronounced glycogen depletion and hypoglycemia after birth [46]. Thus, DEPTOR plays an important role in allowing an appropriate fasting-to-feeding metabolic transition by affecting mTORC1 activity.

Over the last years, several groups have studied the impact of mTORC1 activation on liver physiology and metabolism. The vast majority of the studies were based on the conditional deletion of *Tsc1*, a component of TSC that negatively regulates mTORC1. Using this model, it was first shown that the chronic activation of mTORC1 increases liver weight and blocks ketogenesis upon fasting [2], an effect linked to a defect in the expression of key ketogenic genes, including *Ppara* [2], [6], [47]. Several reports next showed that sustained mTORC1 signaling severely reduces triglyceride deposition in the liver, thus linking hepatic mTORC1 to lipid metabolism [3], [4], [5], [48]. It was also reported that liver-specific *Tsc1* null mice have decreased locomotor activity and body temperature when fasted [2], [4], an effect dependent on an elevation in fibroblast growth factor-21 (FGF21) expression and secretion by the liver [4]. In addition to these metabolic studies, one study showed that chronic activation of hepatic mTORC1 is sufficient to cause hepatocellular carcinomas in mice [49]. Since TSC1 and DEPTOR are both negative regulators of mTORC1, it was expected that some of the phenotypes reported in liver-specific *Tsc1* null mice would also be observed in response to DEPTOR deletion. Surprisingly, DEPTOR loss did not induce any of these phenotypes. Accordingly, Alb-Li-*Deptor*^ko^ mice did not have bigger liver and did not show change in hepatic triglyceride content. We did not observe any impact of DEPTOR deletion on the expression of *Ppara* and *Fgf21* and in the circulating levels of FGF21 (Supplementary Figure 5A–B). Moreover, Li-*Deptor*^ko^ did not develop tumors, even when followed over a period of 12 months (data now shown). Despite being both categorized as inhibitors of mTORC1, major differences exist between DEPTOR and TSC1 that likely explain these different results. First, the level of mTORC1 activation produced from the loss of DEPTOR and TSC1 greatly differs. Here, we show that DEPTOR deletion increases S6 phosphorylation by about 25% in the liver of fasted mice. In sharp contrast, losing TSC1 promotes hepatic S6 phosphorylation by several folds in the same conditions [2], [3], [6], [49]. In addition to generate a more robust impact on mTORC1 effectors, TSC1 loss is recognized to induce severe insulin resistance by promoting mTORC1-mediated feedback inhibition of PI3K [50], [51], [52], [53], [54], [55]. The development of insulin resistance secondary to TSC1 deletion represents a limitation of this model, as it is difficult to delineate the contribution of mTORC1 versus insulin resistance to the phenotype studied. In our case, the loss of DEPTOR did not affect PI3K-Akt/PKB activation. These results likely explain why losing DEPTOR or TSC1 does not lead to the same physiological outcomes. From a conceptual standpoint, these results also underline fundamental differences between these two proteins. Whereas TSC1 is a master switch controlling mTORC1 activity, DEPTOR should be seen as a modulator allowing a better control of mTORC1 when circulating nutrients and growth factors are decreasing. Taking this into account, it would be interesting to compare the metabolic impact of DEPTOR to another negative modulator of mTORC1, namely PRAS40 [17], [18]. Unfortunately, there are currently no studies reporting the impact of liver-specific deletion of PRAS40.

We have previously reported that peripheral overexpression of DEPTOR cell-autonomously promotes adipogenesis [19]. Paradoxically, systemic overexpression of DEPTOR (periphery and brain) was shown to protect mice against the development of obesity by affecting the mediobasal hypothalamic regulation of energy balance [20]. In an attempt to define precisely which neuronal populations of the mediobasal hypothalamus were responsible for the beneficial metabolic effects of DEPTOR, we have overexpressed DEPTOR specifically in proopiomelanocortin (POMC) but found no impact on body weight regulation [43]. Rather, overexpression of DEPTOR in these neurons resulted in liver steatosis and insulin resistance following a high-fat diet [43]. Another group also studied the impact of DEPTOR overexpression *in vivo* by overexpressing the protein in skeletal muscle. In that case, it was reported that DEPTOR overexpression promotes glycolysis and protects mice from developing insulin resistance [44]. Although these results are complex and involve multifaceted functions of DEPTOR in different systems, all these studies showed that DEPTOR overexpression amplifies Akt/PKB signaling by relieving mTORC1-mediated feedback inhibition of PI3K. Here, we provide evidence that deleting DEPTOR enhances mTORC1 activity only during fasting, without impairing PI3K signaling. Our findings strongly argue that endogenous DEPTOR plays marginal roles when circulating nutrients and insulin levels are high, a moment at which mTORC1 is fully active and DEPTOR targeted to the proteasome [16], [37], [38], [39]. Forcing DEPTOR expression in this context reduces but does not fully inhibits mTORC1 activity, which appears to be sufficient to further activate PI3K-Akt/PKB signaling. Thus, the physiological consequences of manipulating DEPTOR expression probably depend on the nutritional status. Moreover, because basal mTORC1 activity differs between cell types and tissues, it is likely that the impact of DEPTOR loss or overexpression will be different depending on the site targeted.

## 5 Conclusion

In conclusion, we report the development of a conditional mouse model allowing the study of the physiological functions of DEPTOR in specific cell types. Using this model, we found that hepatic DEPTOR regulates the metabolic transition to fasting by potentiating mTORC1 inhibition during food restriction. These results highlight the importance of hepatic DEPTOR in fine-tuning liver metabolism during the feeding-to-fasting transition.

## Authors contribution

Conceptualization, AC, MM, ML; Methodology, ML, AC, DMS; Formal Analysis, SML; Investigation, AC, MM, NG, SML, RV, LT, BS, GL, MS, YG; Writing – Original Draft, AC, MM, ML; Writing – Review & Editing, AC, MM, ML; Funding Acquisition, ML, DMS; Supervision, AM, DR, DMS, ML; Resources, AM, DR, DMS, ML.

## Appendix A Supplementary data

Figure S1 **Whole-body DEPTOR null female mice do not display any gross physical abnormalities.** (A) Body weight and (B) tissue weight of whole-body *Deptor* wild-type and knockout female mice. The data are expressed as the mean ± SEM for n = 6–10. (C) Blood metabolites measured in *Deptor* wild-type or knockout female mice. Blood was collected from mice that were fasted overnight. The data are expressed as the mean ± SEM for n = 6–10. Figure S1

Figure S2 **Liver-specific DEPTOR null mice do not exhibit any gross physical and metabolic abnormalities**. (A) Body weight and (B) liver weight in Alb-Li-*Deptor*^wt^ and Alb-Li-*Deptor*^ko^ mice. (C) Plasma insulin, (D) triglycerides and (E) NEFA in Alb-Li-*Deptor*^wt^ and Alb-Li-*Deptor*^ko^ mice. Blood was collected from mice that were fasted for 12 h. The data are expressed as the mean ± SEM for n = 5. * indicates p < 0.05 versus Alb-Li-*Deptor*^wt^ mice. (F) Glycemia measured in fed and fasted Ad5-Li-*Deptor*^wt^ and Ad5-Li-*Deptor*^ko^ mice. Blood was collected from mice at given time. The data are expressed as the mean ± SEM for n = 9. * indicates p < 0.05 versus Alb-Li-*Deptor*^wt^ mice. (G) Food intake following a 12 h fast in Ad5-Li-*Deptor*^wt^ and Ad5-Li-*Deptor*^ko^ mice. The data are expressed as the mean ± SEM for n = 5. * indicates p < 0.05 versus Alb-Li-*Deptor*^wt^ mice. (H) Body weight and (I) liver weight in in Ad5-Li-*Deptor*^wt^ and Ad5-Li-*Deptor*^ko^ mice. The data are expressed as the mean ± SEM for n = 8–9. Figure S2

Figure S3 **Liver-specific DEPTOR null mice do not exhibit change in neoglucogenic gene expression.** Quantitative RT-PCR analyses of liver samples isolated from fasting Alb-Li-*Deptor*^wt^ and Alb-Li-*Deptor*^ko^ mice. The data are expressed as the mean ± SEM for n = 7–9. Figure S3

Figure S4 **Loss of DEPTOR does not affect glycogen deposition in the muscle and minimally affects hepatic mitochondrial biogenesis and expression of components of the respiratory chain**. (A) Muscle glycogen content in Alb-Li-*Deptor*^wt^ and Alb-Li-*Deptor*^ko^ mice. Mice were fasted for 12 h and sacrificed. The data are expressed as the mean ± SEM for n = 5. (B) Hepatic glycogen content in Alb-Li-*Deptor*^wt^ and Alb-Li-*Deptor*^ko^ mice fasted for 12 h and refed for the indicated time. The data are expressed as the mean ± SEM for n = 4–5. (C) *Gys* mRNA expression measured by quantitative RT-PCR analyses in liver samples isolated from fasting Alb-Li-*Deptor*^wt^ and Alb-Li-*Deptor*^ko^ mice. The data are expressed as the mean ± SEM for n = 7–9. (D) Hepatic triglyceride content in Alb-Li-*Deptor*^wt^ and Alb-Li-*Deptor*^ko^ mice. Mice were fasted for 12 h and sacrificed. The data are expressed as the mean ± SEM for n = 5. (E) mtDNA content in Alb-Li-*Deptor*^wt^ and Alb-Li-*Deptor*^ko^ mice. Mice were fasted for 12 h and sacrificed. The data are expressed as the mean ± SEM for n = 5. (F) mtDNA content in control or DEPTOR knockdown AML12 cells. The data are expressed as the mean ± SEM for n = 6. (G) Western blot presenting the expression of proteins of the respiratory chain in the liver of fasted Alb-Li-*Deptor*^wt^ and Alb-Li-*Deptor*^ko^ mice. (H) Western blot presenting the expression of proteins of the respiratory chain in AML12 cells. Figure S4

Figure S5 **DEPTOR loss does not affect *Ppara* expression and FGF21 production.** (A) *Ppara and Fgf21* mRNA expression measured by quantitative RT-PCR analyses in liver samples isolated from Alb-Li-*Deptor*^wt^ and Alb-Li-*Deptor*^ko^ mice. Mice were fasted for 12 h. The data are expressed as the mean ± SEM for n = 7–9. (B) Circulating levels of FGF21 measured in fasted Alb-Li-*Deptor*^wt^ and Alb-Li-*Deptor*^ko^ mice. The data are expressed as the mean ± SEM for n = 8–9. Figure S5
