# Supplementary figures and images for: Loss of hepatic DEPTOR alters the metabolic transition to fasting
Source: Mol Metab. 2017 Feb 17;6(5):447–58. doi: 10.1016/j.molmet.2017.02.005 (PMC5404102; doi:10.1016/j.molmet.2017.02.005)

Figure S1

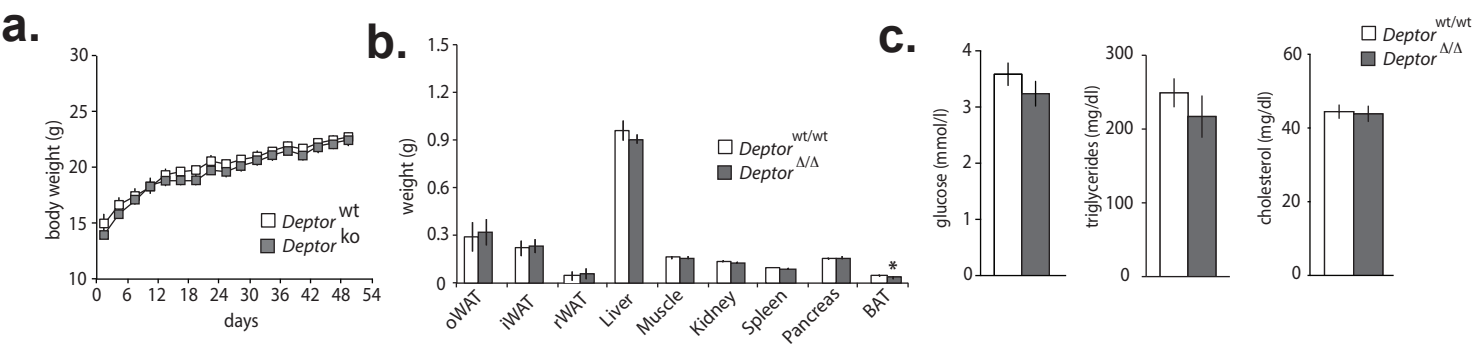

Supplement: Figure S1 — Whole-body DEPTOR null female mice do not display any gross physical abnormalities. (A) Body weight and (B) tissue weight of whole-body Deptor wild-type and knockout female mice. The data are expressed as the mean ± SEM for n = 6–10. (C) Blood metabolites measured in Deptor wild-type or knockout female mice. Blood was collected from mice that were fasted overnight. The data are expressed as the mean ± SEM for n = 6–10. [file mmc1.pdf]

Figure S2

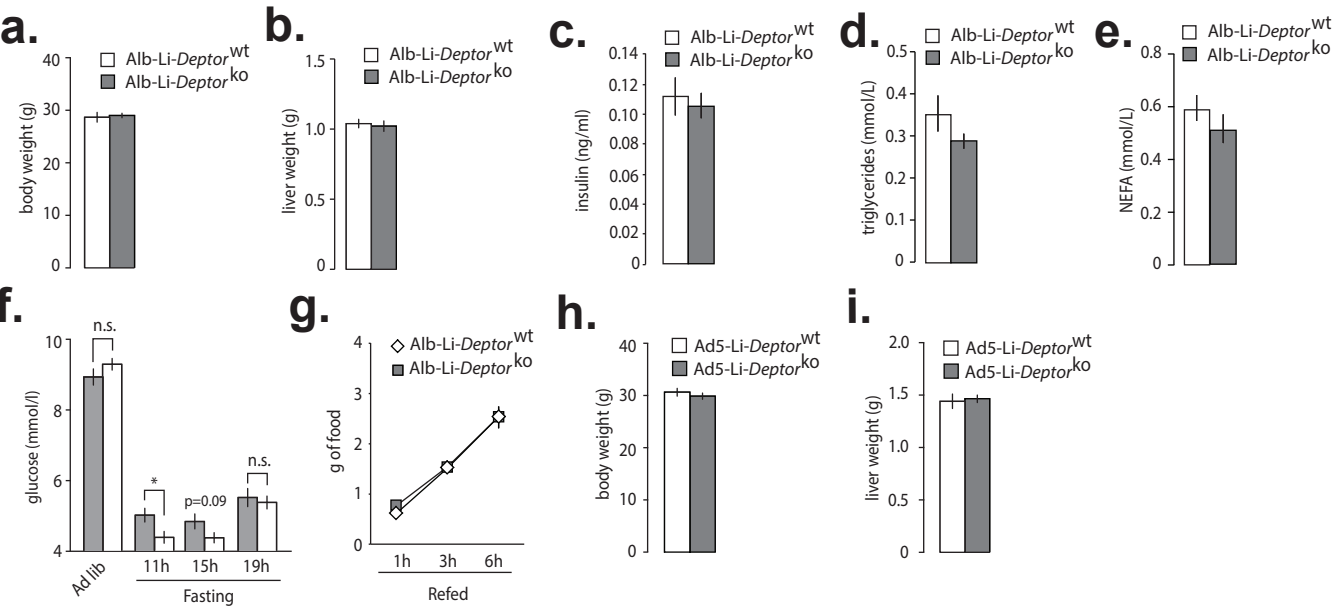

Supplement: Figure S2 — Liver-specific DEPTOR null mice do not exhibit any gross physical and metabolic abnormalities. (A) Body weight and (B) liver weight in Alb-Li-Deptorwt and Alb-Li-Deptorko mice. (C) Plasma insulin, (D) triglycerides and (E) NEFA in Alb-Li-Deptorwt and Alb-Li-Deptorko mice. Blood was collected from mice that were fasted for 12 h. The data are expressed as the mean ± SEM for n = 5. * indicates p < 0.05 versus Alb-Li-Deptorwt mice. (F) Glycemia measured in fed and fasted Ad5-Li-Deptorwt and Ad5-Li-Deptorko mice. Blood was collected from mice at given time. The data are expressed as the mean ± SEM for n = 9. * indicates p < 0.05 versus Alb-Li-Deptorwt mice. (G) Food intake following a 12 h fast in Ad5-Li-Deptorwt and Ad5-Li-Deptorko mice. The data are expressed as the mean ± SEM for n = 5. * indicates p < 0.05 versus Alb-Li-Deptorwt mice. (H) Body weight and (I) liver weight in in Ad5-Li-Deptorwt and Ad5-Li-Deptorko mice. The data are expressed as the mean ± SEM for n = 8–9. [file mmc2.pdf]

Figure S3

a.

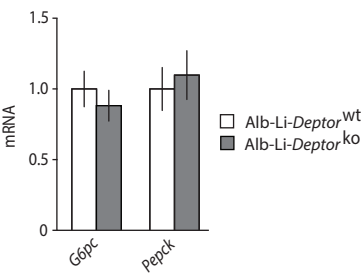

Supplement: Figure S3 — Liver-specific DEPTOR null mice do not exhibit change in neoglucogenic gene expression. Quantitative RT-PCR analyses of liver samples isolated from fasting Alb-Li-Deptorwt and Alb-Li-Deptorko mice. The data are expressed as the mean ± SEM for n = 7–9. [file mmc3.pdf]

Figure S4

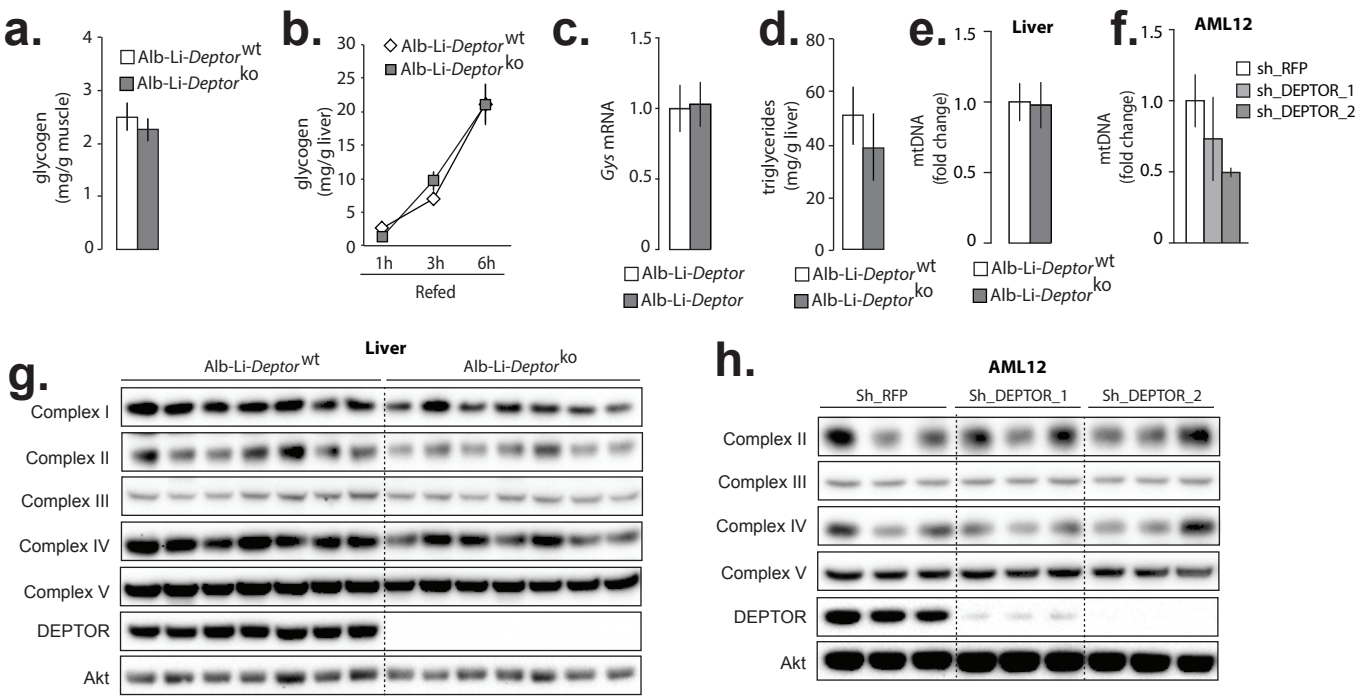

Supplement: Figure S4 — Loss of DEPTOR does not affect glycogen deposition in the muscle and minimally affects hepatic mitochondrial biogenesis and expression of components of the respiratory chain. (A) Muscle glycogen content in Alb-Li-Deptorwt and Alb-Li-Deptorko mice. Mice were fasted for 12 h and sacrificed. The data are expressed as the mean ± SEM for n = 5. (B) Hepatic glycogen content in Alb-Li-Deptorwt and Alb-Li-Deptorko mice fasted for 12 h and refed for the indicated time. The data are expressed as the mean ± SEM for n = 4–5. (C) Gys mRNA expression measured by quantitative RT-PCR analyses in liver samples isolated from fasting Alb-Li-Deptorwt and Alb-Li-Deptorko mice. The data are expressed as the mean ± SEM for n = 7–9. (D) Hepatic triglyceride content in Alb-Li-Deptorwt and Alb-Li-Deptorko mice. Mice were fasted for 12 h and sacrificed. The data are expressed as the mean ± SEM for n = 5. (E) mtDNA content in Alb-Li-Deptorwt and Alb-Li-Deptorko mice. Mice were fasted for 12 h and sacrificed. The data are expressed as the mean ± SEM for n = 5. (F) mtDNA content in control or DEPTOR knockdown AML12 cells. The data are expressed as the mean ± SEM for n = 6. (G) Western blot presenting the expression of proteins of the respiratory chain in the liver of fasted Alb-Li-Deptorwt and Alb-Li-Deptorko mice. (H) Western blot presenting the expression of proteins of the respiratory chain in AML12 cells. [file mmc4.pdf]

Figure S5

a.

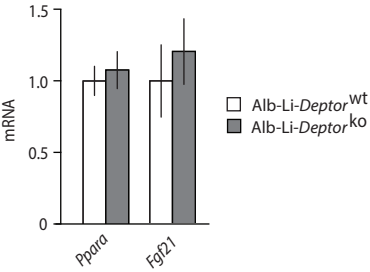

b.

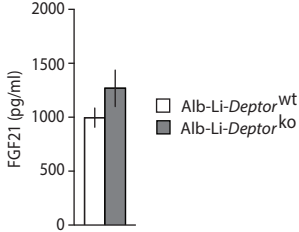

Supplement: Figure S5 — DEPTOR loss does not affect Ppara expression and FGF21 production. (A) Ppara and Fgf21 mRNA expression measured by quantitative RT-PCR analyses in liver samples isolated from Alb-Li-Deptorwt and Alb-Li-Deptorko mice. Mice were fasted for 12 h. The data are expressed as the mean ± SEM for n = 7–9. (B) Circulating levels of FGF21 measured in fasted Alb-Li-Deptorwt and Alb-Li-Deptorko mice. The data are expressed as the mean ± SEM for n = 8–9. [file mmc5.pdf]
